# Ethnomedicinal applications of animal species by the local communities of Punjab, Pakistan

**DOI:** 10.1186/s13002-018-0253-4

**Published:** 2018-08-15

**Authors:** Muhammad Altaf, Muhammad Umair, Abdul Rauf Abbasi, Noor Muhammad, Arshad Mehmood Abbasi

**Affiliations:** 1 Department of Zoology, Women University of Azad Jammu and Kashmir, Bagh, Pakistan; 2 School of Agriculture and Biology, Shanghai Jiao Tong University, Shanghai, China; 3 Statistical Wing, Department of Mathematics, Women University of Azad Jammu and Kashmir, Bagh, Pakistan; 4 Department of Fisheries and Aquaculture, Punjab, Pakistan; 5 Department of Environment Sciences, COMSATS University Islamabad, Abbottabad Campus, Abbottabad, Pakistan

**Keywords:** Animal species, Traditional therapies, Local communities, Pakistan

## Abstract

**Background:**

Different species of animals are being utilized in traditional therapies by various cultures for a long time and such uses still exist in folk medicine. The present study aimed to document animal-based traditional therapies used by the local communities of Jhelum and Lahore districts of the Punjab province, Pakistan.

**Methods:**

Field surveys were conducted in 2015–2016 in six different sites of the study areas. Data were collected through semi-structured interviews and face to face conversation with local informants.

**Results:**

The ethnomedicinal uses of 57 species of animals including mammals, birds, fish, reptiles, amphibian, and invertebrates (30, 25, 25, 7, 3.5, and 3.5%, respectively) were documented. Meat, oil, brain, fats, milk, eggs, and skin were the most utilized body parts. *Ovis orientalis punjabiensis*, *Francolinus francolinus*, *Sperata sarwari*, *Channa punctata*, *Oreochromis niloticus*, *Ctenopharyngodon idella*, *Cyprinus carpio*, *Labeo rohita*, and *Carassius auratus* were reported for the first time to treat human diseases, i.e., allergy, epilepsy, fever, joint pain, and backache, to act as aphrodisiac, and to enhance memory. *Streptopelia decaocto* and *S. tranquebarica* were the most frequently utilized species with highest frequency of citation (32 for each). *Columba livia* depicted highest fidelity level and used value of 92.86% and 0.89, respectively.

**Conclusions:**

Being agro-pastoralists, the inhabitants of Jhelum possess more traditional knowledge compared to Lahore. The present study could be important for conservation and sustainable use of animal biodiversity in this region. Additionally, detailed study on chemical profiling and bioactivities may lead to animal-based novel drug discovery.

## Background

Different body parts of wild and domestic animals are being utilized since ancient time in the prevention and protection of human health disorders [1] and such therapeutics are termed as zootherapy [2]. Zootherapy has profound history with wide geographical distribution. It has been reported that Chinese used earthworms to treat diseases nearly 4000 years ago [3]. Over, 1500 animal species have been documented in Traditional Chinese Medicines, which are used to treat various diseases [4]. Around 15–20% of the Ayurvedic medicines is of animals’ origin [5], and more than 500 species of invertebrates are used to cure both common and complex illnesses in India [6].

Petting, watching, stroking, and working with different animal species can be relaxing, can lower heart beat and stroke, and can be physically beneficial [7]. Chemicals from animals and plant species have been a part of human culture to improve health [8]. Certainly, animals as therapeutic agents have been contributing significantly to the prevention and treatment of health disorders across the globe [9]. It has been estimated that 8.7% of the essential chemicals used in protective drugs are animal based [10]. Because of immunological, analgesic, antibacterial, diuretic, anesthetic, and anti-rheumatic properties, insects are essential components of modern drugs [11]. Chitosan, derived from exoskeleton of insects, is used as an anticoagulant, to lower cholesterol levels in the blood and to repair tissues [12]. Potential anticancer drugs have been isolated from the wings and legs of Asian sulfur butterflies and Taiwanese stag beetles [13].

Pharmaceutical industries are testing many animal species for drug discovery [14]. The best-known example is of snake venom that inhibits angiotensin-converting enzyme (ACE), responsible for the conversion of angiotensin hormone from an inactive precursor, which causes narrowing the blood vessels and raises blood pressure [15]. Similarly, a number of compounds having a defensive role such as biogenic amines, steroids, alkaloids, and peptides have been reported in the secretions of amphibians [16]. These chemical substances possess diverse pharmacological effects including cardiotoxic, myotoxic, and neurotoxic activities [17].

Wildlife is an important but poorly known source to treat many infectious diseases, particularly the zoonotic disorders [18]. The trade in wildlife for food, medicine, and products and as pets, among other uses, involves hunting and the sale of animals of many species [19–22]. Ethnomedicinal information collected form aboriginal peoples contribute significantly to recognize novel biological resources for commercial utilization, mainly in pharmaceutical industries [23, 24]. In addition, expansion of modern medicines is based on traditional knowledge of indigenous communities. Consequently, documentation of the traditional knowledge of indigenous people is imperative, because in the recent era modern drug development has greatly been affected due to loss of socioeconomic and cultural characteristics of local communities around the globe [4]. Pakistan has a rich diversity of animals including 195 “species of mammals” [25], 668 “species of birds” [26], 195 “species of herptiles” [27], over 1000 “species of marine and fresh water fishes”, and 5000 “species of insects” [28]. A number of these species are being utilized in traditional health care. However, ethno-medicinal uses of animal species have rarely been recorded. Furthermore, we imagine that ethnozoological knowledge of local communities residing in settled areas is threatened due to increasing population, urbanization, and industrialization, which should be documented before depletion. Therefore, the current survey aimed to assess and document ethnomedicinal uses of animal species among the local communities of two districts Jhelum and Lahore of the Punjab province, Pakistan.

## Methods

### Field sites

**Fig. 1:**
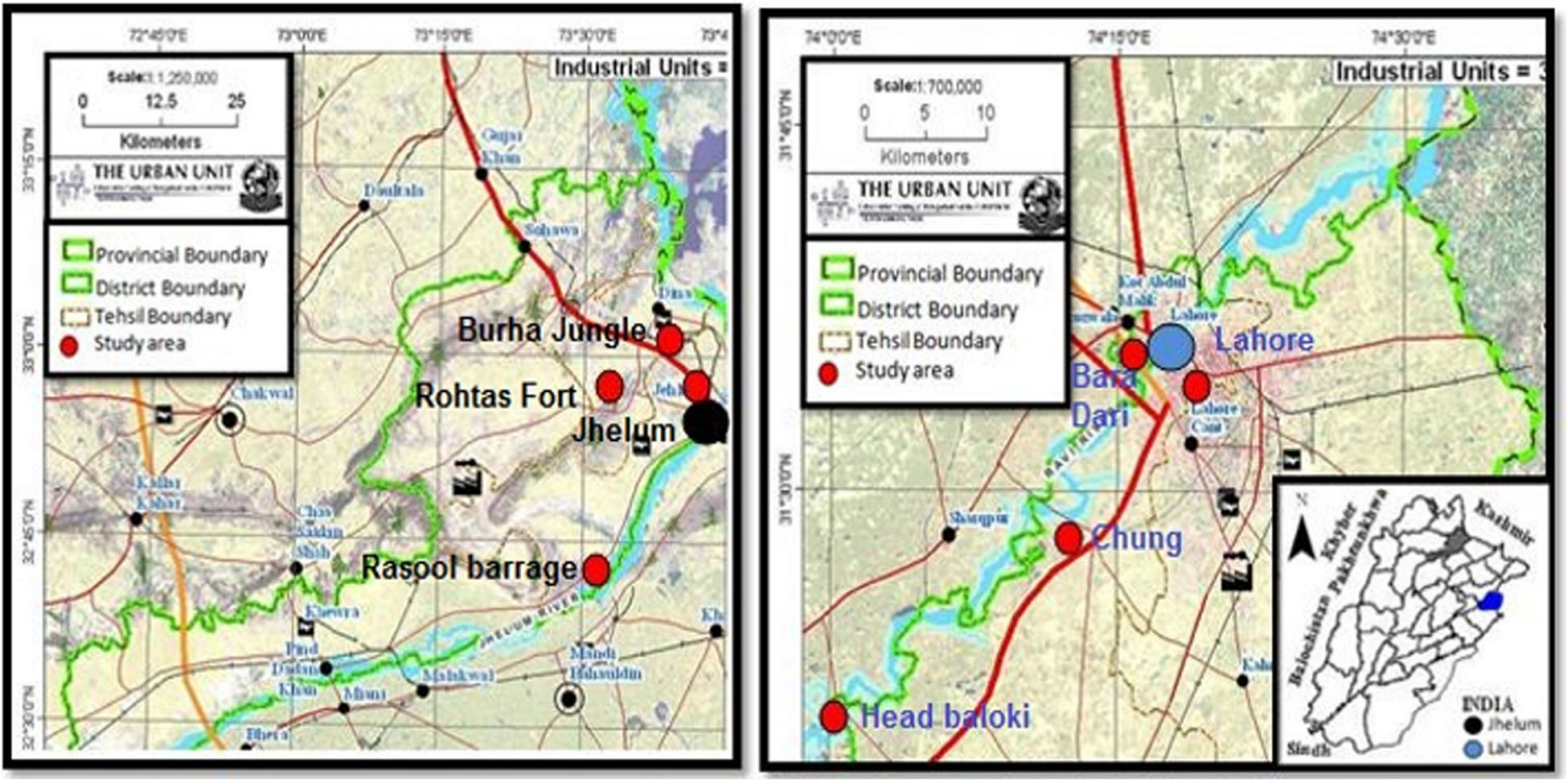
Map of the study area

Ethnozoological survey was conducted in 2012 and 2016 in four sub-areas of district Jhelum: Jhelum city, Burha Jungle, Rohtas fort, and Rasool barrage, and four sub-areas of district Lahore: Lahore city, Bara dari, Chung, and head Baloki (Fig. 1).

District Jhelum is located towards North of the river Jhelum and surrounded by district Rawalpindi in the North, Azad Kashmir from the East, Gujrat and Sargodha districts in the South, and district Chakwal from the West [29, 30]. Total population of the district is 1.223 million, out of which 71% live in rural areas and the rest of 29% is urban population [31]. Approximately, 98.5% of the population is Muslim, while among minorities Christians are in majority with 1%. Awan, Syed, Kashmiri, Ghakar, Gujjar, Mughal, Jat, and Janjua are the major ethnic groups in this area. Jhelum is a semi-mountainous area, receives 880 mm mean annual rain fall, and has 23.6 °C average annual temperature. The inhabitants of Jhelum are agro-pastoralists because of their origin from different parts of Azad and Jammu Kashmir (India). Inhabitants in the rural areas of Jhelum live in mud and brick houses and speak Punjabi and/or Pothohari language. Agriculture, livestock, and mining are the main sources of income. Khewra salt mine in Jhelum is the world’s second largest salt mine [30, 32]. District Jhelum is rich in animal diversity, particularly due to the Mangla dam on the Jhelum River. This wetland is an excellent habitat for bird species. Scientists [33] reported 336 species of birds including 153 breeding residents, 115 winter visitors, 15 summer visitors, 39 passage visitors, and 14 occasionally recorded species. The Mangla water reservoir also provides habitat and food for a variety of fish species. The mammalian fauna of the area is mainly of Oriental origin. The main mammalian species belong to *Canis*, *Herpestes*, *Lepus*, *Lutra*, *Manus*, *Mus*, *Rattus*, *Suncus*, *Sus*, *Viverricula*, and *Vulpes* genera [34].

Lahore is also called as the heart of Pakistan, because it is the hub for culture in the Punjab region and Pakistan as a whole. District Lahore is located towards the North of the river Ravi and is surrounded by Kasur district in the South, district Sheikhupura in the North West, whereas in the East and North-East it is bordered by Indian Punjab [29, 35]. According to a recent survey, the total population of the district is 11.33 million. More than 40% of its inhabitants are below the age of 15 and the same percentage of the population is literate. Almost all inhabitants of this area live in an urban environment. Lahore is the second largest city in Pakistan after Karachi and 32nd largest district in the world. Around 94% of the population is Muslim comprising Sunni and Shia while 6% are minorities, i.e., Christians, Hindus, and Sikhs [36]. Majority of the people in the district speak Punjabi, however, in different dialects, which make it a diverse speaking population area. Urdu and English languages are also spoken and understand by a large number of the population. The average annual rain fall in Lahore district is about 490 mm. The winter in Lahore is cooler than Jhelum with temperature ranging from 1.2 to 15 °C whereas in summer temperature rises up to 46 °C [30, 35]. Nevertheless, Lahore district has greater extension in its urban area. However, still ancient shrines, gardens, cemeteries, traditional buildings, canals, and roads are present, which are the home of many birds, amphibians, and reptiles. In previous records, more than 240 bird species have been documented in Lahore; however, this number is restricted to 85 species now, due to urbanization [37]. Changa Manga forest near Lahore is a wildlife hotspot including wild boar, deer, jackal, nilgai, Asiatic wild cat, and Old World vultures [38]. Increasing population, urbanization, and industrialization depicted great impact on the floral and faunal diversity of Lahore district. Virgin areas of the district supporting natural flora and fauna have been devastated and replaced by buildings, roads, and industries. A greater part of the native flora has been replaced by alien plant species, which is gradually removing the fauna. Such invasion of exotic species poses risk to the biotic uniqueness of the local ecosystems and traditional knowledge of local communities [39]. In a study conducted in Nishtar and Wagah towns of Lahore, about 49 species of birds were reported [40]. In a recent survey, 3 amphibian and 15 reptilian species were reported from Kasur near Lahore [41].

### Data collection and analysis

Before the field survey, prior consent was taken from the Department of Zoology, Women University of Azad Jammu and Kashmir, Bagh, Pakistan. In addition, we also followed the ethical guidelines of the International Society of Ethnobiology (http://www.ethnobiology.net) during the study. Questionnaires and semi-structured interviews were conducted to document ethnomedicinal uses of animal species. Data were collected from 100 informants in each district including farmers, herdsmen, hunters, teachers, and traditional health practitioners (Table 1). Informants were selected based on their knowledge on medicinal uses of animal species. Mammals, birds, herptiles, and fish species were recognized using field guides “*Mammals of Pakistan*” [42, 43], “*Birds of Pakistan*” [44, 45], “*Amphibian and Reptiles of Pakistan*” [46], and “*Freshwater Fishes of Pakistan*” [47]. Data on ethnomedicinal uses and cultural values were analyzed using various indices such as frequency of citation (FC), use value (UV), relative importance (RI), fidelity level (FL), similarity index (SI), principal component analysis (PCA), and cluster analysis (CA).

**Table 1:** Ethnographic data of local informants

Variables	Demographic categories	Jhelum	Lahore
Gender	Male	100	99
	Female	0	1
Experience	Health practitioners	19	18
	Farmer	35	45
	Teachers	31	15
	Herdsman	10	12
	Hunters	5	9
Age group	20–30	5	9
	31–40	27	21
	41–50	36	34
	51–60	26	21
	Above 60	11	15
Education	Post-graduate	0	1
	Graduate	12	7
	SSC	20	23
	Primary	34	45
	Illiterate	34	24
Residence	Rural	77	65
	Urban	23	35
Religious background	Muslim	98	99
	Non-Muslim	2	1

#### Frequency of citation (FC)

FC is the number of informants who reported medicinal uses of each species.

#### Relative importance (RI)

The relative importance (RI) of animal species cited by the informants is calculated as follows:

$$ \mathrm{RI}=\mathrm{PP}+\mathrm{AC} $$

where PP is the number of pharmacological properties (reported specific ailments) attributed to a species divided by the maximum number of properties attributed to the most resourceful species (species with the highest number of properties). AC is the number of ailment categories treated by a given species divided by the maximum number of ailment categories treated by the most resourceful species. A value of 2 is the highest possible value for relative importance (RI) indicating the most versatile species with the greatest number of medicinal properties [19].

#### Fidelity level (FL)

The value of FL highlights the percentage of informants who declare the similar uses of some species [48]. It is calculated by

$$ \mathrm{FL}\ \left(\%\right)=\left(\mathrm{Np}/N\right)\times 100 $$

where Np is the informants’ number, calming medicinal use of animal species contrary to a specific disease and *N* is the total number of informants.

#### Use value (UV)

The use value (UV) can be used to prove the relative importance of species. UV of a species is calculated using the equation:

$$ \mathrm{UV}=\sum U/n $$

where UV is the use value of a species, *n* is the number of citations per species, and *U* is the number of informants [49, 50].

#### Similarity index (SI)

Similarity index (SI) is calculated using the formula:

$$ \mathrm{SI}={M}_{\mathrm{s}}/{M}_{\mathrm{t}}\ \left(0\ \mathrm{SI}\le 1\right) $$

where *M*_s_ is the similar number of medicinal applications in present and previous research records of a species and *M*_t_ is the total number of medicinal applications in present research records.

### Statistical analysis

Data on traditional uses of animal species to treat various ailments were statistically analyzed using principal component analysis (PCA) and cluster analysis (CA) methods with the help of statistical software as described earlier [51].

## Results and discussion

### Ethnography

The data were collected from male Muslims (98%) and non-Muslims (2%) in Jhelum, with both genders male (99%) and female (1%) with religion as Muslims (99%) and non-Muslims (1%) in Lahore. The respondents have different occupations as health practitioners, farmer, teachers, herdsman, and hunters from both districts. They have the education as primary, illiterate, SSC, graduate, and post-graduate. Most of the respondents have age 41–50, while lowest age respondents belong to 20–30-year age in both districts. Most of the respondents belong to almost two thirds from rural and the other from the urban area in both districts Jhelum and Lahore (Table 1).

### Ethnomedicinal application of animal species

The inhabitants of the study area possess significant traditional knowledge and use different animals to treat various health disorders. Out of 57 animal species, 55 were used in Jhelum and 54 in Lahore to treat nervous disorders, paralysis, joint pain, asthma, and fever and to heal wounds and used as aphrodisiacs (Table 2). Nutritional deficiency, lack of a hygienic environment, and social evils may attribute to the high prevalence of these diseases in both study areas. *Bufo stomaticus* (Indus valley toad), *Heteropneustes fossilis* (scorpion cat-fish), *Lissemys punctate andersoni* (Indian flap-shelled turtle), and *Oligochaeta* spp. (earthworms) were the commonly utilized species in Lahore. Conversely, the inhabitants of Jhelum use *Hoplobatrachus tigerinus*, (Indian bullfrog), *Naja naja naja*, (black cobra), *Pteropus giganteus* (Indian flying fox bat), and *Bagarius bagarius* (bag arid catfish) to treat various diseases.

**Table 2:** Comparison of medicinal uses of animal species

Sr. #	Scientific name, Common name, Vernacular name, Code	District Jhelum (J)					District Lahore (L)					Previous reports			
		PU/MA	Disease treatment	FC	UV	RI	PU/MA	Disease treatment	FC	UV	RI	Diseases treated	Ref.	SI	
														J	L
Amphibian															
1	*Bufo stomaticus* Lütken, Indus Valley toad, Maidani daddo, BS			0	0	0	Skin/T	Allergy	7	0.29	0.3	Thelitis, dermatitis, decubitus wounds, ripened abscess, brunchest, pneumonia, bolianerengia	[79, 80]	0	0
2	*Hoplobatrachus tigerinus* (Daudin), Indian Bullfrog, Wada daddo, HT	Fat/T	Sexual enhancement	2	0.38	0.39						Diarrhea, dysentery, cold and cough, burn, wound, acidity	[64, 81–83]	0	0
			Muscular pain	2											
			Joint pain	2											
			Headache	2											
Reptiles															
3	*Lissemys punctata andersoni* Webb, Indian Flap-shelled Turtle, Hara Kachupra, LPA				0.00	0.00	Carapace/T	Allergy	1	0.29	0.3	Rashes, burns, asthma, lung diseases, cough, tuberculosis, Diarrhea, indigestion, malaria fever, diabetes, urinary obstruction, arthritis, Bronchitis, menorrhagia, sexual dysfunction, wounds, dermatitis, acne, piles	[73, 74, 84]	0	0
							Fat/T	Sexual enhancement	1						
								Backbone pain	1						
								Epilepsy	2						
								Cession	1						
							Bile/T	Strangulation	1						
4	*Laudakia agrorensis* (Stoliczka), Monitor lizard, Goh, Wada Kirla, LA	Fat/ T	Burn	4	0.22	0.38				0.00	0.00	Cough, fever, jaundice, burn, joint pain, skin disease, arthritis, malaria, sexual stimulant	[80, 81, 83, 85–87]	0.51	0
			Sexual male power	5											
5	*Saara hardwickii* (Gray), Indus Valley spiny-tail ground lizard, Sanda, UH	Fat/T	Sexual enhancement	10	0.84	1.56	Fat/ T	Sexual enhancement	10	0.73	1.21	Increase sexual power	[88]	0.25	0.25
								Weakness	2						
			Muscular pain	5				Ear pain	5						
			joint pain	5				Backbone pain	5						
			Head-ach	5											
6	*Naja naja* (L.), Black cobra, Kala Naag, NNN	Skin/ T	Sharpen eye side	10	0.50	0.39	Fat/ T	Muscular pain	3	0.53	0.90	Muscular pain, arthritis and sexual weakness, leprosy, cancer	[82, 83, 88]	0	0.33
								Sexual weakness	10						
		Oil/ T	Snake bite	2			Oil/ T	Snake bite	2						
7	*Echis carinatus sochureki* Stemmler, Sind Valley saw snake viper, Daba sap, ECH	Oil/ T	Snake bite	2	0.29	0.38				0.00	0.00	Snake bite	[88]	0.50	0
			Sexual enhancement	5											
Mammals															
8	*Lepus nigricollis dayanus* Blanford, Desert hare, Jungli saya/Jungli khargush, LND	Meat/ O	Paralysis	2	0.87	1.18	Hair/ T	Burning sensation	2	0.78	0.92	Tonic, chicken pox, wheezing, stomach and joint pain, high blood pressure, Asthma, burning sensation, paralysis.	[63, 64, 73, 74, 77, 85, 89–91]	0.67	0.50
			Weakness	4			Meat/ O	Paralysis	20						
			Asthma	4				Asthma	10						
9	*Hystrix indica* Kerr., Indian crested porcupine, Kanday wali say, HCR	Fat/ T	Skin infection	3	0.36	0.77	Fat/ T	Skin infection	2	0.50	0.60	Skin infection, rheumatic pain, colic, boiled, stomach-ache, foot mouth disease, easy delivery of a child, premenstrual pain, weakness and muscle fatigue, asthma	[64, 77, 80, 82, 84, 90, 92–94]	0.08	0.14
			Joint pain	10				Joint pain	8						
10	*Pteropus giganteus* (Brün.), Indian flying fox bat, Chamgadar, PGI	Fat/ T	Enhance sexual male power	7	0.29	0.38	Fat/ T	Enhance sexual male power	9	0.33	0.30	Asthma, bronchitis, enhance sexual power	[73, 74, 77, 85, 95]	0	0.20
11	*Rattus rattus* (L.), House rat, Wada Choha, RR	Fat/ T	Joint pain	8	0.13	0.38	Fat/ T	Joint pain	7	0.29	0.30	Convulsions, semen enhancement, wounds healing, joint pain	[74, 77, 81, 95, 96]	0.20	0.20
12	*Ovis orientalis* punjabiensis Lydekker, Urial, Heeran, OO	Meat/ O	Enhance power	18	0.83	0.40	Fat/ T	Joint pain	10	0.73	0.91			0	0
								Backbone pain	2						
								Sexual enhancement	10						
13	*Hemiechinus collaris* (Gray), Long eared desert hedgehog, Chotay kanday ali say/Kandyari Choha, HCO	Fat/ T	Joint pain	9	0.33	0.77	Fat/ T	Joint pain, Backbone pain	10	0.30	0.60	Rheumatic pain, body ache	[77]	0.50	0.50
			Backbone pain	1											
14	*Canis aureus* L., Golden jackal, Gidar, CAA	Fat/ T	Skin infection	7	0.29	0.38	Fat/ T	Skin infection	8	0.25	0.30	Rheumatic pain, body ache	[63, 64, 74, 77, 81, 86, 91, 93, 97]	1.0	1.0
15	*Herpestes javanicus* (E. Geoffroy Saint-Hilarie), Small Indian mongoose, Neola, HJ	Fat/ T	Sexual power	8	0.27	0.77	Fat/ T	Sexual power	9	0.22	0.30	Sexual power, impotence by males	[77, 86]	1.0	1.0
			Backbone pain	3											
16	*Camelus dromedarius* L., Dromedary, Ount, CD	Milk/ O	Hepatitis B and C	10	0.75	0.78	Milk/ O	Joint pain	2	0.67	1.50	Acidity, hepatitis B and C, malaria, cold, coughs, stannic pain, migraine headache, lumbago (for buffalo)	[77, 88, 92, 98–100]	0.33	0.5
			Cancer	10				Diabetes	1						
								Hepatitis B and C	10						
								Allergy	1						
								Cancer	14						
17	*Capra aegagrus hircus* (L.), Goat, Bakri, CAH	Milk/ O	enhance energy sexual power	20	0.86	0.80	Milk/ O	Enhance sexual power	10	0.90	0.33	Fever, eye tonic, tonsillitis, asthma, tuberculosis, menstrual disorder, toothache, anemia, cough, dysentery, bronchitis, jaundice, diarrhea, blindness, joint pain, sexual enhancement, rhinitis, skin blisters	[73, 74, 77, 81, 83, 88, 89, 95, 96, 98, 101–103]	0.33	0.33
							Testis/ O	Enhance sperm production	10						
		Meat/ O	Fever	8			Bone soup/ O	Heal wound	9						
18	*Bos taurus* L., Cattle, Gay, BT	Ghai and fat/T	Feet wounds	10	0.73	1.55	Ghai and fat/ T	Feet wounds	4	0.82	1.21	Fever, bone fever, memory loss, paralysis, asthma, stomach ache, gastritis, diarrhea, eye infection, sore throats, tuberculosis, pesticide, measles, wound, cough, body pain, poison effect, acne and facial pimples, blood cancer, appetite stimulant, malaria, hysteria	[63, 64, 66, 77, 83–85, 89, 90, 96, 98, 100, 103–107]	0.43	0.38
		Milk and meat/O	Body pain, Fever	10			Milk and meat/ O	Body pain, Fever	6						
							Ghai and milk/O	Poison effect	2						
							Testis and milk/ O	Enhance the sperm production.	2						
		Ghai and milk/O	Poison Effect	2			Testis and milk/ O	Enhance the sperm production	10						
19	*Bubalus bubalis* (L.), Buffalo, Mujh, BB	Milk and turmeric/O	Wound	4	0.74	3.08	Milk and turmeric/ O	Wound	1	0.72	2.39	Pain, wound, jaundice, ascites, rheumatic pain, weakness, osteoporosis, thrombosis, improves heart strength, pre-menstrual pain, injury	[73, 74, 77, 80, 81, 85, 89, 95, 108–110]	0.11	0.09
		Milk and almond/ O	Enhance physical and mental health	1			Milk and almond/ O	Enhance physical and mental health	2						
		Colostrum/ O	Enhance immunity	2			Colostrum/ O	Enhance immunity	2						
		Milk, Fenugreek seed, turmeric, white piper grind and mixed all/ O	Diabetes	1			Milk, Fenugreek seed, turmeric, white piper grind and mixed all/ O	Diabetes	2						
		Milk mixed with grind water caltrop/ O	Enhance sexual power	2			Milk mixed with grind water caltrop/ O	Enhance sexual power	2						
							Milk mixed with grind water caltrop/ O	Enhance sexual power	1						
		Milk mixed with grind water caltrop/ O	Enhance sexual power	2											
		O = Milk mixed with grind seeds of dates/ O	Joint pain	2			Milk mixed with grind seeds of dates/ O	Joint pain	3						
			Heart diseases	3				Heart diseases	2						
			Stones of bladder	2				Stones of bladder	3						
			Stones of spleen	1				Stones of spleen	1						
			Enhance sexual power	3				Enhance sexual power	4						
20	*Manis crassicaudata* É.. Geoffroy, Indian pangolin, Sipa/ Sipple, MC	Scale/T	enhance sexual Power	9	0.33	0.39	Scale/ T	enhance sexual Power	9	0.22	0.30	Feet swelling, piles, blood pressure, head ach, asthma, anti-haemorrhoidal, warts, ear pain, angina, back pain, heals bone inflammation, anti-poison, heals torn veins and arteries, infertility, gastro-intestinal disorders, safe parturition, stomach ulcers, rheumatism and fibroid, sexual power	[73, 74, 82, 84, 89, 91, 93, 110–112]	1.0	0.05
21	*Homo sapien*s L., Human, Insan, HS	Saliva/T	Herpes	4	0.33	0.77	Saliva/ T	Herpes	4	0.25	0.59	Eye infections, wound, hiccup, herpes, ear pain, conjunctivitis, eye pain, antiseptic in	[67–69, 77, 80, 85, 91, 97, 101, 113]	1.0	1.0
		Urine/T	Ear pain	2			Urine/ T	Ear pain	6						
22	*Ovis aries* L., Sheep, Bairh, OA	Milk/T	Skin burn and cracks	16	0.69	1.17	Milk/ T	Skin burn and cracks	2	0.77	1.21	Edema, fractures, joint pain, sterility, flu, skin burn and crack, muscular pain, swellings, weakness, rheumatism, arthritis, swells, breast infection, headache, brain diseases, phlegm, dizziness, night blindness, heart failure, epilepsy, scabies	[19, 59, 64, 66, 70, 73, 74, 77, 100, 103, 107, 110, 113–116]	0.75	0.75
							Soup/ O	Flu	4						
							Meat/ O	Weakness and joint pain	10						
		Meat/O	Weakness and joint pain	10			Testis/ O	Enhance sperm production	10						
23	*Felis chaus* Schreber, Jungle cat, Jungli billi, FC	Fat/T	Joint pain	8	0.50	0.39	Fat/ T	Joint pain	8	0.63	0.30	Leucoderma, joint pain	[74, 77]	1.0	1.0
24	*Felis domesticus* Erxleben, Domestic cat, Billi, FD	Fat/T	Joint pain	9	0.33	0.39	Fat/ T	Joint pain	9	0.44	0.30	Fever, arthritis, Rheumatic pain, skin infections, Goiter	[77, 81, 100, 101]	1.0	1.0
25	*Oryctolagus cuniculus* (L.), Domestic rabbit, Khargush/Saya, OC	Meat/ O	Paralysis	10	0.72	0.78	Meat/ O	Paralysis	12	0.78	0.61	Bronchial diseases, stomachache, burn, weakness	[77, 88, 100, 102, 104]	0.30	0.30
			Asthma	8				Asthma	6						
Birds															
26	*Passer domesticus* (L.), House Sparrow, Chiri, PD	Meat/O	Weakness	5	0.75	0.78	Meat/ O	Sexual power	5	0.80	0.61	Increase sexual desire, aphrodisiac, allergy, paralysis, impotency, gas trouble, constipation, Chickenpox, weakness, fever, delay dentition (child)	[64, 74, 77, 83, 88, 93, 94]	0.50	0.50
			Energy	4				Energy	6						
			Fatigue	2				Fatigue	6						
			Fever	9				Weakness	9						
27	*Gallus gallus* (L.), Domestic chicken, Murghi, Kukri, GG	Egg mixed with milk/O	Weakness	10	0.86	1.57	Egg/ O	Breast cancer	9	0.89	2.41	Sprains, strains, nourishing food, eye-each, BP, bronchitis, hemorrhoids, diabetes, burst furuncles, asthma, indigestion, jaundice, diabetes, sinusitis, to ease birth, shortness of breath, bronchitis, nervous problems, rheumatism, stuffy nose, weak bones, flu, weakness, sore throat, furuncle, burns, night blindness, optic infection, evil eye	[19, 64, 66–68, 77, 82, 83, 85, 90, 93, 95, 101–103, 107, 115–117]	0.30	0.10
			Low blood pressure	5				Weight loss	1						
								Eye sight	5						
		Meat/O	Fever	10				Deficiency of protein	3						
			Cold	10				Energy	2						
								Cold	2						
								CNS	1						
								Bones and teeth nourishment	6						
28	*Columba livia* Gmelin, Blue rock pigeon, Jangli kabotar, CL	Meat/ O	Paralysis	20	0.89	0.42	Meat/ O	Paralysis	22	0.93	0.33	Menorrhagia, Bronchitis, puberty in young girls, paralysis, epilepsy, anemia, infertility, Menorrhagia, abscess	[73, 74, 77, 81, 82, 86, 88, 96–98, 102, 117]	0.33	0.33
			Weakness	4				Weakness	20						
			Enhance energy	4				Enhance energy	20						
29	*Coturnix coturnix* (L.), Common quail, Batera, CCO	Brain/ O	Enhance memory	22	0.81	0.81	Brain/ O	Enhance memory	10	0.83	0.94	Skin diseases, anemia, body weakness, enhance memory, sexual power, fever	[64, 73, 74, 77]	0.40	0.40
		Meat/ O	Enhance energy	1			Meat/ O	Enhance energy	8						
			sexual power	5				sexual power	5						
			against cold	5				against cold	3						
30	*Francolinus francolinus* (L.), Black francolin, Kala tittar, FFR	Meat/ O	Enhance energy	10	0.77	0.80	Meat/ O	Enhance energy	1	0.80	0.92	Bronchitis, weakness	[77, 88]	0	0
			Sexual power	10				Sexual power	10						
			Paralysis	6				Paralysis	2						
			Against cold	4				Against cold	5						
31	*Anas platyrhynchos f. domesticus*, Domestic duck, Batakh, APD	Meat/ O	Enhance energy	10	0.84	1.18	Meat/ O	Fever	5	0.88	1.81	Weak eye-side, weakness, low blood pressure	[77]	0.30	0.10
		Egg/ O	Fever	8				Enhance energy	6						
			Weak eye side	14			Egg/ O	Weak eye side	1						
								Increase protein	1						
								CNS	1						
								Strengthened bones and teeth	2						
32	*Streptopelia tranquebarica* (Hermann), Red turtle dove, Surakh totru, STR	Meat/ O	Early maturity in young female	18	0.44	0.39	Meat/ O	Early maturity in young female	30	0.50	0.31	Maturity in girls	[77]	1.00	0
33	*Streptopelia decaocto* (Frivaldszky), Indian ring dove, Kogi/Ghogi, SDE	Meat/ O	Early maturity in young female	16	0.44	0.39	Meat/ O	Early maturity in young female	32	0.50	0.31	Maturity in girls, sexual tonic	[77, 94]	1.00	0
34	*Streptopelia orientalis* (Latham), Oriental turtle dove, Totru, SOR	Meat/O	Early maturity in young female	14	0.43	0.39	Meat/ O	Early maturity in young female	14	0.50	0.31	Maturity in girls	[77]	1.00	0
35	*Spelopelia senegalensis* (L.), Little brown dove, Chhoti tutru/Chhoti kogi, SSE	Meat/ O	Early maturity in young female	15	0.38	0.39	Meat/ O	Early maturity in young female	13	0.46	0.30	Maturity in girls	[77]	1.00	0
36	*Athene brama* (Temminck), Spotted owlet, Ullo, ABR	Blood/ T	Enhance male power and treat sexual weakness	21	0.79	0.41	Blood/ T	Enhance male power and treat sexual weakness.	24	0.75	0.32	Rickets, cough, sexual weakness	[73, 77]	0.50	0.50
37	*Acridothere ginginianus* (Latham), Bank myna, Lali, AGI	Meat/ O	Whooping cough	15	0.47	0.39	Meat/ O	Whooping cough	15	0.40	0.30			0	0
38	*Anas platyrhynchos* L., Mallard, Nilsir, APL	Meat/ O	Paralysis	7	0.53	0.77	Meat/ O	paralysis	5	0.47	0.60	Erectile dysfunction, scarlet fever, body strength, weakness, paralysis	[66, 73, 74, 77, 96]	0.5	0.25
							Egg/ O	Eye sight	3						
								Enhance energy	2						
		Egg/ O	Enhance energy	8				Protein	5						
39	*Aquila nipalensis* Hodgson, Tawny eagle, Baaz, ARN	Fat/ T	Breast swelling and pain	17	0.53	0.39	Fat/ T	Breast swelling and pain	17	0.47	0.31	Chest pain, breast swelling	[59, 77]	0.50	0.50
40	*Upupa epops* L., Common hoopoe, Hud-hud, UEP	Meat/ O	Kidney problems	9	0.44	0.39	Meat/ O	Kidney problems	9	0.56	0.30	Gall bladder stone, kidney problems	[77, 93]	1.00	1.00
Fishes															
41	*Rita rita* (Hamilton), Rita, Khaga, RRI	Brain/ O	Enhance memory	4	0.81	1.17	Brain/ O	Enhance memory	4	0.86	1.21	Joint pain	[88]	0.21	0
		Meat/ O	Enhance energy	5			Meat/ O	Enhance energy	5						
			Sexual power	2				Sexual power	2						
		Oil/ O	Energy	5			Oil/ O	Reduce overweight	5						
			Against cold	5				Energy	5						
			Joint pain	1				Against cold	2						
42	*Sperata seenghala* (Sykes), Giant river catfish, Singhari, SPSA	Brain/ O	Enhance memory	5	0.72	1.16	Brain/ O	Enhance memory	4	0.67	1.20			0	0
		Meat/ O	Enhance energy				Meat/ O	Enhance energy	5						
			Sexual power	5				Sexual power	2						
		Oil/ O	Energy				Oil/O	Reduce overweight	5						
			Against cold					Energy	2						
			Joint pain					Against cold	2						
43	*Channa punctata* (Bloch), Spotted snakehead, Dola, CPU	Brain/ O	Enhance memory	1	0.84	1.18	Brain/ O	Enhance memory	5	0.81	1.22	Blood purification, appetite, malaria control, body pain, corn or calves	[118–120]	0	0
		Meat/ O	Enhance energy	6			Meat/ O	Enhance energy	5						
			Sexual power	3				Sexual power	10						
								Reduce overweight	5						
		Oil/ O	Energy				Oil/ O	Energy	5						
			Against cold	5				Against cold	1						
			Joint pain	1											
44	*Channa marulius* (Hamilton), Bullseye snakehead, Sap machli, CMA	Brain/ O	Enhance memory	3	0.81	1.17	Brain/ O	Enhance memory	3	0.86	1.21	Increase sex power of male increase hemoglobin level, rheumatic pain	[88, 118, 121, 122]	0.17	0.17
		Meat/ O	Enhance energy	2			Meat/ O	Enhance energy	2						
			Sexual power	5				Sexual power	5						
		Oil/ O	Energy	3			Oil/ O	Reduce overweight	3						
			Against cold	2				Energy	2						
			Joint pain	6				Against cold	6						
45	*Oreochromis niloticus* (L.), Baringo tilapia, Chirra machhli, OAU	Brain/ O	Enhance memory	3	0.50	1.54	Brain/ O	Enhance memory	3	0.56	1.49	Abscesses, sharpen sight, carbuncle, scorpion bite	[123]	0	0
		Meat/ O	Enhance energy	2			Meat/ O	Enhance energy	2						
			Sexual power	3				Sexual power	5						
			Scorpion bite	1				Reduce overweight	3						
		Oil/ O	Energy	1			Oil/ O	Sharpens eye sight	2						
			Against cold	6				Energy	6						
			Joint pain	1				Against cold	1						
46	*Labeo calbasu* (Hamilton), Black rohu, Kalbans, LCA	Brain/ O	Enhance memory	1	0.50	1.15	Brain/ O	Enhance memory	3	0.57	1.49	Increase energy and memory, galactagogue	[121]	0.33	0.33
		Meat/ O	Enhance energy	2			Meat/ O	Enhance energy	2						
			Sexual power	3				Sexual power	5						
		Oil/ O	Energy	1			Oil/ O	Reduce overweight	1						
			Against cold	1				Increase lactation in mother	1						
			Joint pain	6				Energy and cold	2						
47	*Ctenopharyngodon idella* (Steindachner), Gardd carp, Grass carp, CID	Brain/ O	Enhance memory	2	0.73	1.16	Brain/ O	Enhance memory	3	0.67	1.20	Against cold	[124]	0	0
		Meat/ O	Enhance energy	2			Meat/ O	Enhance energy	2						
			Sexual power	1				Sexual power	5						
		Oil/ O	Energy	1			Oil/ O	Reduce overweight	2						
			Against cold	1				Energy	2						
			Joint pain	8				Against cold	1						
48	*Cyprinus carpio* L., Aischgrund carp, Gulfam, CCA	Brain/ O	Enhance memory		0.68	1.16	Brain/ O	Enhance memory	3	0.74	1.20	Erysipelas, lumbago, CNS	[123]	0	0
		Meat/ O	Enhance energy	2			Meat/O	Enhance energy	2						
								Sexual power	5						
			Sexual power	1				Reduce overweight	3						
		Oil/ O	Energy	1			Oil/ O	Energy	2						
			Against cold	3				Against cold	4						
			Joint pain	10											
49	*Cirrhinus mrigala* (Hamilton), Mrigal carp, Marakhi, CMR	Brain/ O	Enhance memory	1	0.73	1.17	Brain/ O	Enhance memory	3	0.77	1.21	Joint pain, reduce weight	[88]	0.17	0.17
		Meat/ O	Enhance energy	2			Meat/ O	Enhance energy	2						
			Sexual power	1				Sexual power	5						
		Oil/ O	Energy	3				Reduce overweight	3						
			Against cold	3			Oil/ O	Energy	2						
			Joint pain	12				Against cold	6						
50	*Labeo rohita* (Hamilton), Roho labeo, Raho, LRO	Brain/ O	Enhance memory	1	0.88	1.19	Brain/ O	Enhance memory	3	0.85	1.22	Urine Problem, stomach ache, weakness, rheumatic pain, Gastric	[80, 88, 118]	0	0
		Meat/ O	Enhance energy	12			Meat/ O	Enhance energy	2						
			Sexual power	1				Sexual power	15						
		Oil/ O	Energy	1				Reduce overweight	3						
			Against cold	3			Oil/ O	Energy	2						
			Joint pain	15				Against cold	6						
51	*Carassius auratus* (L.), Goldfish, Sanhari, CAU	Brain/ O	Enhance memory	1	0.63	1.16	Brain/ O	Enhance memory	3	0.68	1.20			0	0
		Meat/ O	Enhance energy	6			Meat/ O	Enhance energy	2						
			Sexual power	3				Sexual power	5						
		Oil/ O	Energy	1				Reduce overweight	3						
			Against cold	1			Oil/ O	Energy	2						
			Joint pain	7				Against cold	4						
52	*Gibelon catla* (Hamilton), Catla, Thaila, CACA	Brain/ O	Enhance memory	1	0.70	1.17	Brain/ O	Enhance memory	3	0.6	1.2	Increase energy and memory, galactagogue, rheumatic pain	[88, 121]	0.33	0.33
		Meat/ O	Enhance energy	12			Meat/ O	Enhance energy	2						
			Sexual power	2				Sexual power	15						
		Oil/ O	Energy	1				Reduce overweight	3						
			Against cold	1			Oil/ O	Energy	2						
								Against cold	2						
			Joint pain	10											
53	*Wallago attu* (Bloch), Boal, Mali, WAT	Brain/ O	Enhance memory	1	0.74	1.17	Brain/ O	Enhance memory	3	0.70	1.21	Joint pain, liver tonic, blood dysentery and piles	[67, 113, 125]	0.17	0.17
		Meat/ O	Enhance energy	1			Meat/ O	Enhance energy	2						
			Sexual power	13				Sexual power	10						
			Liver diseases	1				Reduce overweight	3						
		Oil/ O	Energy	1			Oil/ O	Energy	2						
			Against cold	3				Against cold	3						
			Joint pain	3											
54	*Bagarius bagarius* (Hamilton), Bagarid catfish, Foji Khaga, BBA	Brain/ O	Enhance memory	1	0.81	1.18	Brain/ O	Enhance memory	3	0.85	1.51	Body burns, stomach pain, body pain	[86, 118]	0.37	0.17
		Meat/ O	Enhance energy	2			Meat/ O	Enhance energy	2						
			Sexual power	10				Sexual power	10						
		Oil/ O	Energy	4				Reduce overweight	3						
			Against cold	1			Oil/ O	Energy	2						
			Joint pain	9				Against cold	2						
55	*Heteropneustes fossilis* (Bloch), Scorpion cat-fish, Singhi, HF	Brain/ O	Enhance memory	1	0.74	1.16	Brain/ O	Enhance memory	3	0.79	1.20	Sting, joint pain, increase hemoglobin level and fever, pain, wound healing	[80, 83, 98, 114]	0.17	0
		Meat/ O	Enhance energy	1			Meat/ O	Enhance energy	2						
			Sexual power	8				Sexual power	5						
		Oil/ O	Energy	1				Reduce overweight	3						
			Against cold	1			Oil/ O	Energy	2						
			Joint pain	7				Against cold	2						
Invertebrates															
56	*Apis mellifera* L., Honey Bee, Shahd makhi, AME	Honey mixed with grind cinnamon/ O	Cold	10	0.89	5.39	Honey mixed with grind cinnamon/ O	Cold	1	0.83	5.95	Dark spots, bronchitis, skin lightening, cough, fever, cataract, burn, sexual impotence, cold, flu, aging, sore throat, shortness of breath, arthritis, tuberculosis, constipation	[19, 59, 66, 67, 94, 96, 100–103, 115]	0.13	0.09
								Cough	1						
			Cough	1				Acidity	1						
			Acidity	3				Obesity	1						
			Obesity	1				Control blood pressure	1						
			Control blood pressure	1				Muscle pain	2						
								Belly pain	1						
			Muscle pain	5				Antimicrobial	3						
			Belly pain	1				Anti-inflammatory	2						
		Fennel mixed with honey/ O	Indigestion	1											
							Honey/T	Hair loss	3						
								Pimple	1						
		Honey/ O	Body pain	3				Insect bite	1						
			Ulcer	1			Grind big raisins, fennel mixed with honey/O	Indigestion	1						
			Allergy	1											
			Tumor	1											
			Enhance immunity	1				Body pain	1						
								Ulcer	1						
		Green tea, fennel, black cardamom, cinnamon mixed with honey/ O	Indigestion	1				Allergy	1						
								Tumor	1						
								Enhance immunity	1						
							Lemon juice, olive oil mixed with honey (in equal quantity)/ O	Kidney stones	1						
							Ghai, egg yolk, mixed with honey/ O	Weak eyesight	1						
							Grind walnut mixed with honey/ O	Stomach diseases, increase energy	1						
							Green tea, fennel, cardamom, cinnamon mixed with honey/ O	Indigestion	1						
57	*Oligochaeta* spp. Earth worm, Gundoya, LTE			0	0.00	0.00	Dry and clean earthworm body take in dry mud pot and pot close with mud and warm it with cow/buffalo dung, now get a ash/ O	Backbone pain	6	0.17	0.30	Wound, impotence	[59, 96]	0	0

Note: O (mean oral), T (topical), PU (parts use), MA (mode of application)

The medicinal uses of *Ovis orientalis punjabiensis* (urial), *Francolinus francolinus* (black francolin), *Sperata sarwari* (giant river catfish), *Channa punctate* (snake head), *Oreochromis niloticus* (baringo tilapia), *Ctenopharyngodon idella* (gradd carp), *Cyprinus carpio* (aischgrund carp), *Labeo rohita* (roho labeo), and *Carassius auratus* (goldfish) were reported for the first time from the study areas. These species are used to treat allergy, epilepsy, fever, joint pain, and backache and to enhance memory and as aphrodisiac. Additionally, they have a zero similarity index with previous reports. However, some species such as *Canis aureus* (golden jackal), *Herpestes javanicus* (small Indian mongoose), *Homo sapiens*, (human), *Felis chaus* (jungle cat), *Felis domesticus* (domestic cat), *Upupa epops* (common hoopoe), *Manis crassicaudata* (Indian pangolin), *Streptopelia tranquebarica* (red turtle dove), *Streptopelia decaocto* (Indian ring dove), *Streptopelia orientalis* (oriental turtle dove), and *Spelopelia senegalensis* (little brown dove) exhibited the highest similarity index (SI = 1) with previous studies.

### Body part(s)

**Fig. 2:**
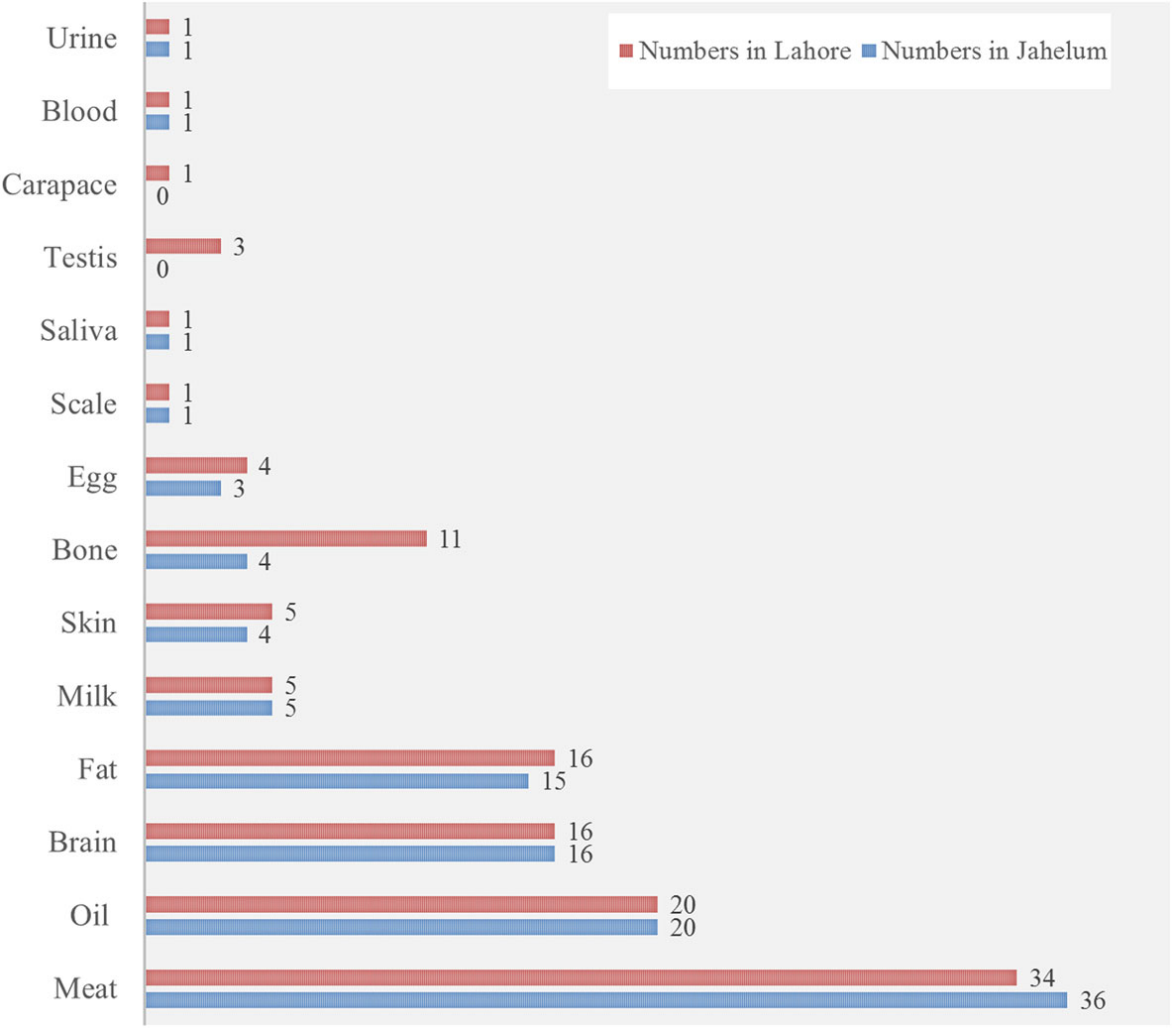
Body parts of animal species used in different recipes

Meat was the most utilized body part and used in 36 recipes in Jhelum and 34 recipes in Lahore (Fig. 2), followed by oil and brain used in 20 and 16 recipes, respectively, in both districts, and fat used in 15 and 16 recipes in Jhelum and Lahore respectively. Milk, skin, bones, eggs, scale, saliva, blood, urine, testis, and carapace were used in less than five recipes. Local inhabitants of Lahore and Jhelum use chopped brains of different species such as common quail, rita, giant river catfish, spotted snakehead, bulls eye snakehead, baringo tilapia, black rohu, gradd carp, aischgrund carp, mrigal carp, Roho labeo, goldfish, catla, boal, bagarid catfish, and scorpion cat-fish to enhance the efficiency of the brain and nervous system. Likewise, testis of *Capra aegagrus hircus* (goat), *Bos taurus* (cattle), and *Ovis aries*, (sheep) are used to enhance the sperm production. However, these uses were more common in Lahore compared to Jhelum. Eggs of *Gallus gallus* (domestic chicken), *Anas platyrhynchos domesticus* (domestic duck), and *Anas platyrhynchos* (mallard) are used to treat fever, cold, weakness, low blood pressure, and weak eye side in Jhelum, while in Lahore they are used to treat breast cancer, weight loss, and cold and to enhance the performance of the CNS and strength of bones and teeth.

**Fig. 3:**
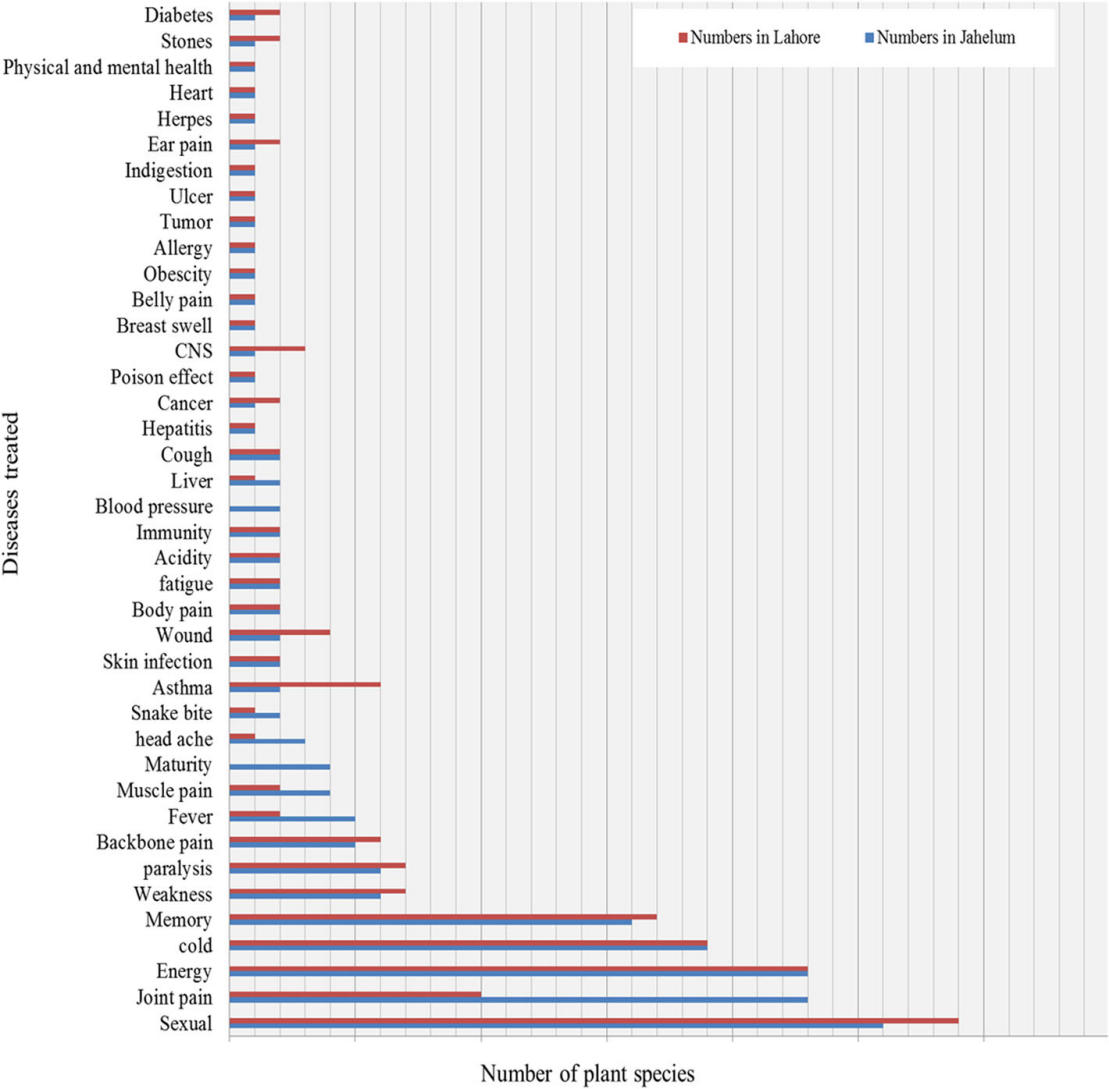
Number of animal species used to treat various diseases in Jhelum and Lahore

**Fig. 4:**
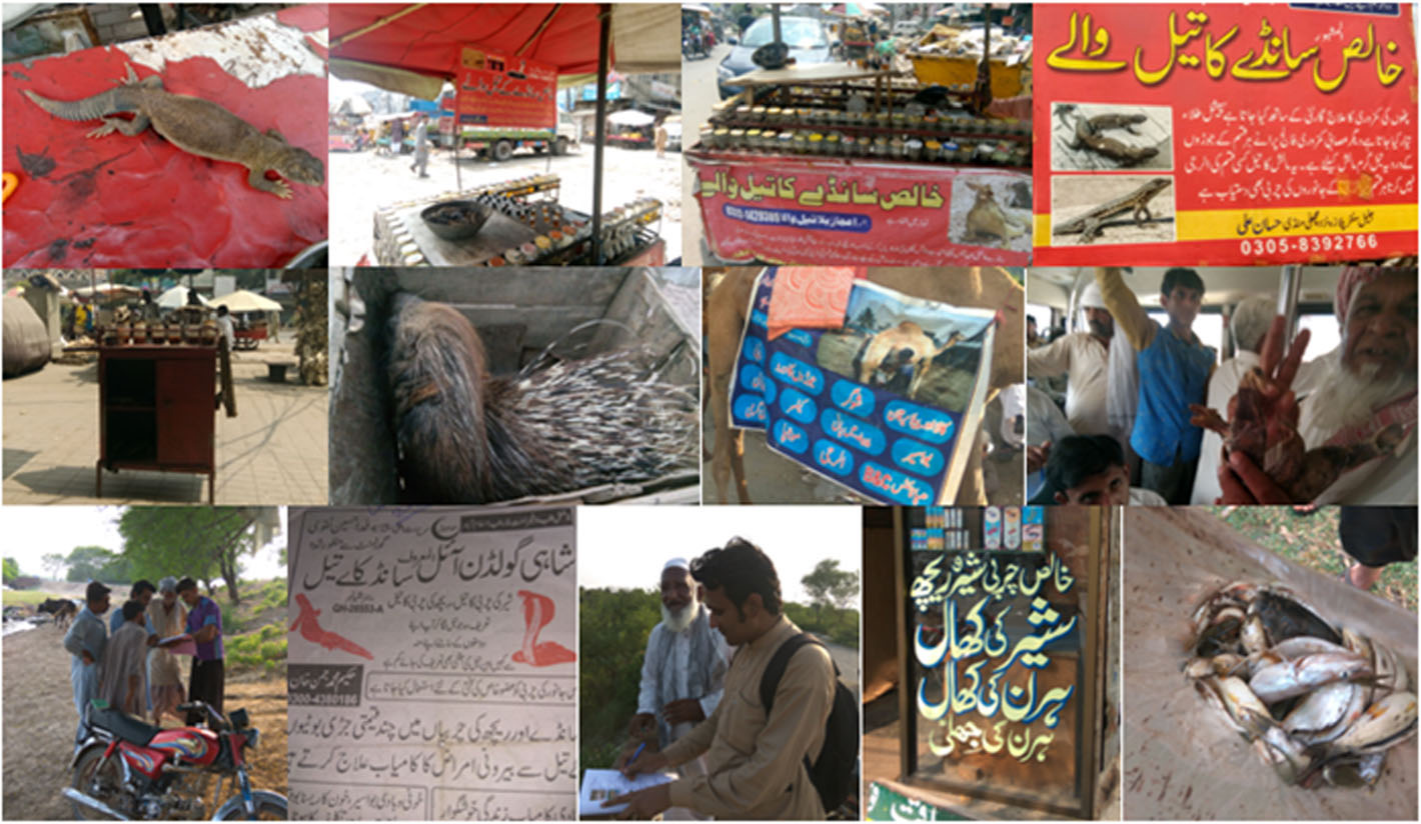
Pictorial views of traditional uses of animal species in the study areas

It has been reported that omega-3 fatty acid in animal fat ore oil reduces inflammation [52]. The present study revealed that inhabitants of the study areas use fat and oil to treat backache, breast swelling, cold, headache, burn, rheumatic pains, snake bite, and skin infections and as a sex stimulant (Figs. 2, 3 and 4). These uses are comparable to previous reports that animal fats or oil are useful in atherosclerosis, neurological disorder, and thrombotic and aging effects [53, 54].

Milk of *Bubalus bubalis* (buffalo), *Bos taurus* (cattle), *Capra aegagrus hircus* (goat), *Camelus dromedarius* (dromedary), *Equus africanus asinus* (donkey), and *Ovis aries* (sheep) is used as a sexual stimulant and antidote; to treat fever, diabetes, blood pressure, backache, and joint pain; for fertility; and to expel kidney stones. It is well known that milk contains high levels of proteins, vitamins, lipids, and minerals, which reduce joint pain, strengthen the body, and increase sexual potency [55–59].

**Fig. 5:**
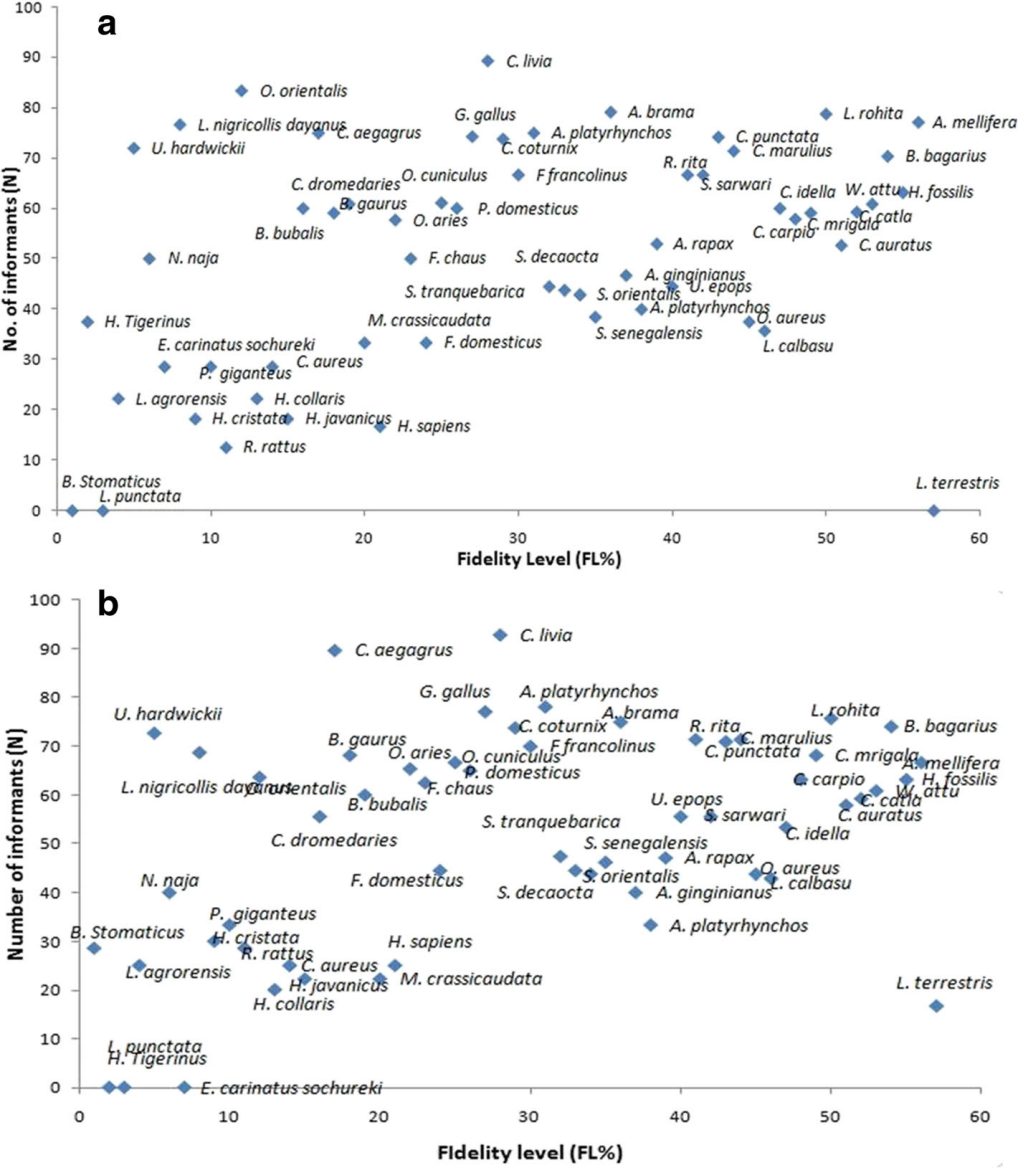
Relationship between informant numbers and the number of application in study sites. (**a**) for Jehlum and (**b**) for Lahore

The inhabitant of Lahore use bone soup of *Capra aegagrus hircus* (goat) to heal internal wounds and fractures (Figs. 4 and 5). This confirms that matrix contains up to 95% collagen fibers, elastic protein, and inorganic minerals like calcium phosphate, which improves fracture resistance [60]. Local communities use scales of Indian pangolin (*Manis crassicaudata*) as a sexual stimulant (in both districts) and to remove hook worms (in Lahore only). The health benefits of Pangolin scales might be due to the presence of different chemical constituents such as cholesterol, stearic acid, volatile oil, minerals, proteins, glycine, isoleucine, leucine, lysine, proline, serine, tyrosine, and valine amino acids among several others [61]. However, due to illegal hunting and extensive use in traditional medicines, Indian pangolin is at the verge of extinction and has been included in “Red Listed” species by the International Union for Conservation of Nature (IUCN) [62].

Human’s urine is used against herpes and to treat ear pain in both districts. It has been known that the urine of cattle, dromedary, sheep, goat, hyrax, rhinoceros, and ass is also useful in the treatment of acne, asthma, anemia, antifungal, burn, back pain, chronic ailment, disinfection, foot diseases, fever, skin infections, TB, mouth infection, syphilis, rashes, CNS, memory loss, throat, and ear and eye infection [20, 63–74]. In addition, urine of dromedary inhibits enhancement of apoptosis, cell proliferation, and control of cyclin-dependent kinase inhibitor p21 [65] and has high resistance against heat and fungal diseases [72].

### Frequency of citation (FC)

Animal species, reported by the maximum number of informants as frequently used to treat various diseases, have high frequency of citation (FC) which ranged from 1 to 32 (Table 2). In different areas of district Lahore, *Streptopelia decaocto* (Indian ring dove) and *S. tranquebarica* (red turtle dove) were reported as the most frequently utilized species for maturity in young girls with FC = 32 each. *Athene brama* (spotted owlet) and *Columba livia* (blue rock pigeon) were also among the commonly used species with FC value of 24 and 22, respectively. In different localities of Jhelum district, *Coturnix coturnix* (common quail) with FC = 22 was the most commonly used species for the enhancement of memory followed *Athene brama* (spotted owlet), *Columba livia* (blue rock pigeon), and *Capra aegagrus* (goat) which have FC values of 21, 20, and 20, respectively, whereas the lowest FC = 1 was calculated for *Homo sapien*s (human) from Jhelum and earthworms from Lahore.

### Fidelity level (FL)

Fidelity level (FL) is used to identify species that are most preferred by the inhabitants to treatment of certain ailments. Animal species with topmost medicinal uses in a particular area have maximum fidelity level [75, 76]. The fidelity levels of animal species used by the inhabitants of Lahore and Jhelum districts are given in Table 3. Among the species reported from Lahore: *Columba livia* (blue rock pigeon) depicted highest FL (92.86%), followed by *Capra aegagrus* (goat) and *Anas platyrhynchos domesticus* (domestic duck) with percentage FL = 89.66 and 78.13, respectively, whereas earthworm had the lowest FL 16.67%. Among the animal species reported from different parts of Jhelum, *Columba livia* (blue rock pigeon), *Ovis orientalis punjabiensis* (urial), and *Athene brama* (spotted owlet) were dominant with maximum percentage fidelity levels of 89.29, 83.33, and 79.71, respectively. However, *Hystrix indica* (Indian crested porcupine) had the lowest FL of 18.18% in Jhelum. The animal species with the highest FL could be used for in-depth chemical profiling and pharmaceutical properties. This will authenticate not only the medicinal worth of these species but could also be useful for novel animal-based drug discovery. Altaf et al. [77] document that the species *B. taurus*, *Oryctolagus cuniculus*, *Ovis aries*, *A. platyrhynchos domesticus*, *G. gallus*, and *P. domesticus* show 100% FL.

**Table 3:** Comparison of fidelity level in the both study areas

S #	Scientific name	Jhelum (FL)	Lahore (FL)
1	*Bufo Stomaticus*	0.000	28.57
2	*Hoplobatrachus Tigerinus*	37.50	0.000
3	*Lissemys punctata andersoni*	0.00	25.00
4	*Laudakia agrorensis*	22.22	0.000
5	*Uromastyx hardwickii*	72.00	72.73
6	*Naja naja naja*	50.00	40.00
7	*Echis carinatus sochureki*	28.57	0.000
8	*Lepus nigricollis dayanus*	76.67	68.75
9	*Hystrix indica*	18.18	30.00
10	*Pteropus giganteus*	28.57	33.33
11	*Rattus rattus*	12.50	28.57
12	*Ovis orientalis punjabiensis*	83.33	63.64
13	*Hemiechinus collaris*	22.22	20.00
14	*Canis aureus*	28.57	25.00
15	*Herpestes javanicus*	18.18	22.22
16	*Camelus dromedarius*	60.00	55.56
17	*Capra aegagrus* hircus	75.00	89.66
18	*Bos taurus*	59.09	68.18
19	*Bubalus bubalis*	60.87	60.00
20	*Manis crassicaudata*	33.33	22.22
21	*Homo sapien*s	16.67	25.00
22	*Ovis aries*	57.69	65.38
23	*Felis chaus*	50.00	62.50
24	*Felis domesticus*	33.33	44.44
25	*Oryctolagus cuniculus*	61.11	66.67
26	*Passer domesticus*	60.00	65.00
27	*Gallus gallus*	74.29	77.14
28	*Columba livia*	89.29	92.86
29	*Coturnix coturnix*	73.81	73.81
30	*Francolinus francolinus*	66.67	70.00
31	*Anas platyrhynchos f. domesticus*	75.00	78.13
32	*Streptopelia tranquebarica*	44.44	47.37
33	*Streptopelia decaocto*	43.75	44.44
34	*Streptopelia orientalis*	42.86	43.75
35	*Spelopeliasenegalensis*	38.46	46.15
36	*Athene brama*	79.17	75.00
37	*Acridothere ginginianus*	46.67	40.00
38	*Anas platyrhynchos*	40.00	33.33
39	*Aquila nipalensis*	52.94	47.06
40	*Upupa epops*	44.44	55.56
41	*Rita rita*	66.67	71.43
42	*Sperata seenghala*	66.67	55.56
43	*Channa punctata*	74.19	70.97
44	*Channa marulius*	71.43	71.43
45	*Oreochromis niloticus*	37.50	43.75
46	*Labeo calbasu*	35.71	42.86
47	*Ctenopharyngodon idella*	60.00	53.33
48	*Cyprinus carpio*	57.89	63.16
49	*Cirrhinus mrigala*	59.09	68.18
50	*Labeo rohita*	78.79	75.76
51	*Carassius auratus*	52.63	57.89
52	*Gibelon catla*	59.26	59.26
53	*Wallago attu*	60.87	60.87
54	*Bagarius bagarius*	70.37	74.07
55	*Heteropneustes fossilis*	63.16	63.16
56	*Apis mellifera*	77.14	66.67
57	*Oligochaeta* spp.	0.000	16.67

### Relative importance (RI)

The relative importance of animal species used by the inhabitant of Lahore and Jhelum districts is mentioned in Table 2. Most of the animal species were found to be highly versatile in their uses such as *Apis mellifera* (honey bee) with RI of 5.95 and 5.39 in Lahore and Jhelum, respectively, followed by *Columba livia* (blue rock pigeon) having RI of 2.4 (Lahore) and 1.6 (Jhelum) and *Uromastyx hardwickii* (spiny-tail ground lizard) and *Heteropneustes fossilis* (singhi) with RI of 1.56 (Jhelum) and 1.5 (Lahore). The maximum RI values might be a sign of high affordability and accessibility of these species in the study areas.

### Use value (UV)

Results of use value (UV) authenticate the relative importance of species or family for a population. This index was anticipated to craft a connotation between each species and the uses allocated to it by analyzing the index in relation to the use groups. Comparative assessment of UV of different animal species among the local communities residing in different parts of Lahore and Jhelum is given in Table 2. Among the reported animal species, the highest UVs of 0.89 and 0.88 were calculated for *Columba livia* (blue rock pigeon) from Jhelum and *Gallus gallus* (domestic chicken) from Lahore, whereas the lowest UVs of 1.6 and 0.12 were attained by earthworm and *Rattus rattus* (house rat) in Lahore and Jhelum in respective order. The high UVs of these species certified their consistent use in the treatment of different diseases. In addition, citation by the maximum number of informants and use reports viewing that these species are well known and commonly utilized for medicinal purpose in the study areas.

### Principal component analysis (PCA) and cluster analysis (CA)

**Fig. 6:**
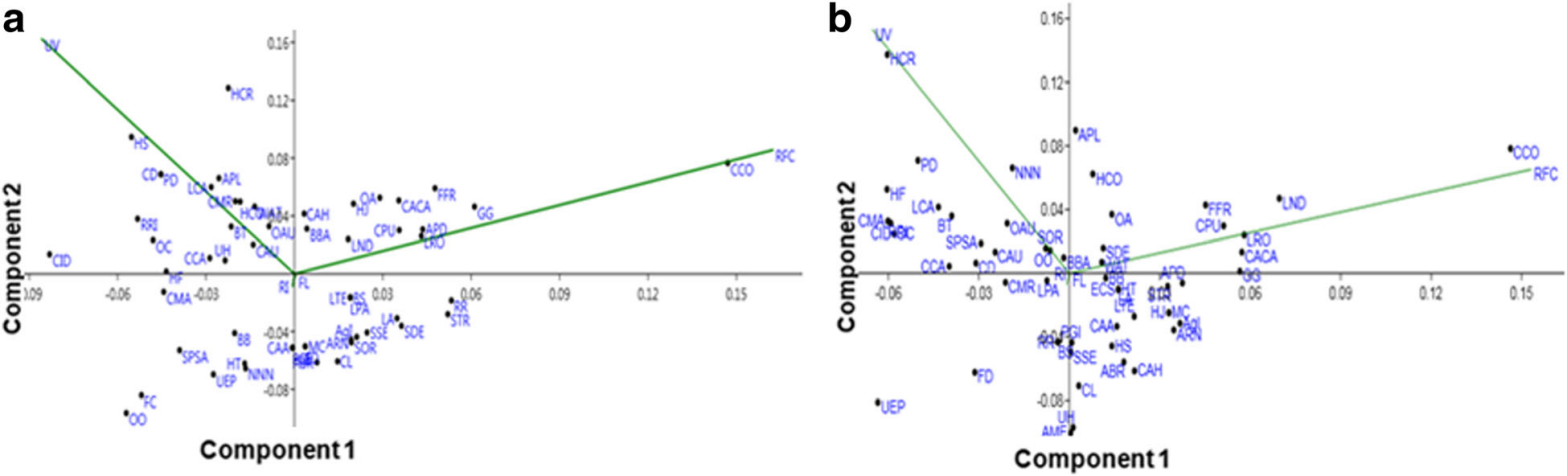
Principal components analysis (PCA) (code are present in Table 2). The positions of the arrows relative to components 1 and 2 show how strongly independent variables (UV, RFC, FL and RI) are correlated with each other from both districts Jhelum (**a**) and Lahore (**b**)

Results of PCA are given in Fig. 6a, b. For district Jhelum, variables loaded onto component 1 include the following: FC (*r* = 0.004), UV (*r* = 0.01), RI (*r* = 0.015), and FL (*r* = 0.999), while on component 2 they included the following: FC (*r* = 0.02), UV (*r* = − 0.038), RI (*r* = 0.998), and FL (*r* = − 0.015). For Lahore district, variables loading onto component 1 were FC (*r* = 0.0067), UV (*r* = 0.011), RI (*r* = 0.016), and FL (*r* = 0.999) and component 2 were FC (*r* = 0.02), UV (*r* = − 0.021), RI (*r* = 0.999), and FL (*r* = − 0.017). The first two axes of the PCA showed 99.9% variation in samples (component 1: 99.9%; component 2: 0.1%) from Jhelum (Fig. 6a) and 99.87% variation in samples (component 1: 99.87%; component 2: 0.119%) from Lahore (Fig. 6b). Each principal component is not correlated with other principal components recorded from Jhelum and Lahore, respectively. Findings are resembled with reported study [78].

**Fig. 7:**
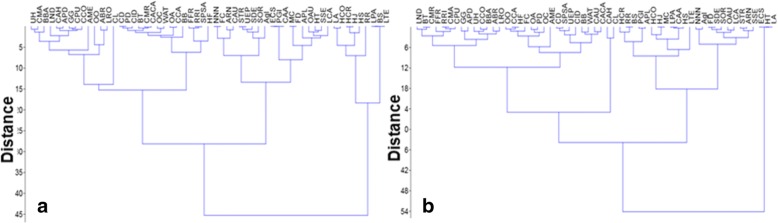
Cluster analysis showing the similarities among species (code are present in Table 2) in different variables (UV, RFC, FL, and RI) of Jhelum (**a**) and Lahore (**b**)

The statistical analysis shows that different groups are present in the cluster analysis, which are differentiated on the basis of values. The statistical analysis shows that two groups are present in the cluster analysis in Jhelum, i.e., group 1 (G1) and group 2 (G2). G1 and G2 have distance/variability of 45 points; G1 has species as LA, HCO, HCR, HJ, HS, RP, LPA, BS, and LTE (code are present in Table 2). G2 has two subgroups, i.e., subgroup 1 (SG1) and subgroup 2 (SG2) have 30 variability points. SG1 was further divided into two groups as SG1A and SG1B (variability = 14 points); SG2 was further divided into two groups as SG2A and SG2B (variability = 15 points) (Fig. 7a, b).The statistical analysis shows that two groups are present in the cluster analysis in Lahore, i.e., group 1 (G1) and group 2 (G2). G1 and G2 have distance/variability of 54 points; G1 has species as ECS, HT, and LA (code are present in Table 2). G2 has two sub groups, i.e., subgroup 1 (SG1) and subgroup 2 (SG2) have 33 variability points. SG1 was further divided into two groups as SG1A and SG1B (variability = 14 points); SG2 was further divided into two groups as SG2A and SG2B (variability = 24 points) (Fig. 7a, b). Findings are resembled with the reported study [78].

## Conclusion

Traditional knowledge of local communities, particularly on the medicinal application of animal species to treat health disorders, indicates their strong association with the surrounding environment. Medicinal uses of herptiles and ichthyo fauna of Pakistan were studied for the first time. Furthermore, application of *O. orientalis punjabiensis*, *F. francolinus*, *S. sarwari*, *C. punctate*, *O. aureus*, *C. idella*, *C. carpio*, *L. rohita*, and *C. auratus* to cure various diseases in humans has rarely been reported before. Our findings provide baseline data that could be valuable in conservation and sustainable use of animal biodiversity in this region. Screening of pharmacological active substances and in vitro or in vivo assessment of biological activities of animal species with maximum FL, UV, RI, and RFM could be important for animal-based novel drugs.
